# Association between Cysticercosis and Neoplasia: A Study Based on Autopsy Findings

**DOI:** 10.1155/2013/895942

**Published:** 2013-10-29

**Authors:** Camila Lourencini Cavellani, Aline Cristina Souza da Silva, Grace Kelly Naves de Aquino Ribeiro, Lívia Ferreira Oliveira, Mara Lúcia Fonseca Ferraz, Vicente de Paula Antunes Teixeira

**Affiliations:** Biological and Natural Science Institute, General Pathology Discipline, Triângulo Mineiro Federal University, Disciplina de Patologia Geral, Rua Frei Paulino No. 30, Bairro Abadia, 38025-180 Uberaba, MG, Brazil

## Abstract

Chronic infections including the cysticercosis induce inflammatory cells to produce free radicals and synthesize carcinogenic toxins. The cells with genetic mutations proliferate in a disorganized manner, leading to the development of neoplasia. The aim of the present study was to demonstrate the relation between cysticercosis and neoplasia. Patients autopsied were divided into 4 groups: patients with neoplasia and cysticercosis (NC), patients with neoplasia only (NN), patients with cysticercosis only (CC), and patients without neoplasia or cysticercosis (WW). Of 2012 autopsy reports analyzed, 0.4 showed NC. In groups CC and NC, the most common location of the parasite was the brain. There was a predominance of three or more cysticerci in groups NC and CC. In the NC group, all had malignant neoplasms, and was predominance of benign neoplasm in NN group. The digestive system was the most frequent neoplasia. By calculating odds ratio, rate of neoplasia in patients with cysticercosis was 0.74. In conclusion, the demographic profile of patients with cysticercosis and neoplasia is similar to that of patients with cysticercosis alone. The incidence of cysticercosis and neoplasia was greater in older patients suggesting that immunosenescence may contribute to development of neoplasia promoted by cysticercosis.

## 1. Introduction

Cysticercosis is a chronic parasitic inflammatory disease caused by the larval form of the *Taenia solium *parasite that elicits inflammatory reactions in both the tissues surrounding the parasite and distant tissues [1, 2]. Cysticercosis is prevalent in several regions of Asia, Africa, and Latin America owing to poor sanitary conditions and socioeconomic conditions [3, 4]. Although in some studies evidence was not provided for the association between infectious and parasitic diseases and neoplasias [5], chronic infections such as cysticercosis have been implicated as the cause of one-third of all newly detected neoplasias worldwide [6–8].

Chronic infections induce inflammatory cells to produce free radicals and synthesize carcinogenic toxins [7, 9, 10]. Parasite-induced immunosuppression via evasive mechanisms, such as the differential expression of antigens, molecular mimicry of human leukocyte antigens, and alterations to the human immune system, is a potential oncogenic effect of cysticercosis [11–14]. Secretion of the metacestode factor by the parasite also contributes to nuclear alterations, such as those that occur in peripheral lymphocytes [15, 16]. Thus, cells with genetic mutations proliferate in a disorganized manner, leading to the possible development of neoplasia [17].

Considering these data, the aim of the present study was to demonstrate the correlation between cysticercosis and the development of neoplasia. Therefore, we also describe the disease characteristics and determine the epidemiologic profile of these patients. 

## 2. Material and Methods

In a descriptive study, we reviewed 2012 reports of autopsies performed between 1970 and 2006 in the Hospital de Clínicas of the Universidade Federal do Triângulo Mineiro, Uberaba, Brazil. Cases were selected and patients were divided into 4 groups: 1, patients with neoplasia and cysticercosis (NC); 2, patients with neoplasia (NN) only; 3, patients with cysticercosis (CC) only; and 4, patients without neoplasia or cysticercosis (WW). Patients were also divided into elderly (aged ≥ 60 years) and nonelderly.

Information was collected from the autopsy files regarding the following parameters: sex, age, ethnic group, presence of cysticercosis, number and location of the cysticerci, presence of neoplasias, and location and nature of the neoplasias. We also divided patients according to the number of cysticerci (1, 2, or ≥3).

Neoplasias were classified according to their benign or malignant nature and divided according to the system in which they were found: digestive, endocrine, gynecological (in women, mammary glands included), reproductive (in men), hematopoietic, nervous, skin, respiratory, urinary, and others. 

For the statistical analysis, we used the Kolmogorov-Smirnov test to determine whether the variables were normally distributed. For analysis of the qualitative variables, we applied the chi-square (*χ* ^2^) test, while for the analysis of the quantitative variables we used the Kruskal-Wallis test. To determine the risk of developing neoplasia in patients with cysticercosis, we calculated the odds ratio (OR) using a confidence interval of 95%. Differences were considered statistically significant when *P* < 0.05.

## 3. Results

The 2012 autopsy reports that were analyzed showed 8 cases (0.4%) of NC, 66 cases (3.3%) of CC, 270 (13.4%) cases of NN, and 1,668 cases (81.9%) of WW.

There was a predominance of Caucasian patients in all groups. Male sex was predominant in groups CC, NN, and WW; however, in group NC, there was no significant sex disparity (Table 1). There was a predominance of elderly patients in the NC group, in which the mean age (66 years) was significantly higher than that of groups CC, NN, and WW (50, 55, and 45 years, resp.).

In the CC and NC groups, the most common location of the cysticercus was the brain, followed by the heart and muscles.

With regard to the number of cysticerci, the NC and CC groups exhibited a predominance of 3 or more cysticerci (50.0% and 47.0%, resp.). 

In the NC group, all neoplasias were malignant, whereas in the NN group the majority of neoplasias were benign (62.5% (*P* = 0.001)). In the NC and NN groups, neoplasias were predominantly located in the digestive system (62.5% and 35.0%, resp.). The next most predominant locations were the respiratory (25%) and gynecological systems (12.5%) for the NC group and the gynecological (14%), hematological (10.0%), and nervous systems (3.7%) for the NN group. 

According to the OR calculation, the risk of developing neoplasia in patients with cysticercosis was 0.748 (0,355; 1,578).

## 4. Discussion

In the present study, 0.4% of the autopsied patients copresented with cysticercosis and neoplasia, which is a lower percentage than that reported by other authors who reported an occurrence of cysticercosis and neoplasia of 20.9% in autopsies [18]. Moreover, all neoplasias were malignant. In an earlier study, 3.7% of living patients presenting with neurocysticercosis also had glioma [19]. This difference in neoplasia and cysticercosis occurrence may be because these studies were conducted using different methodologies.

T lymphocytes, natural killer cells, macrophages, antibodies, and cytokines interleukin- (IL-)2, tumor necrosis factor- (TNF-)*α*, and interferon- (IFN-)*γ* are important defense mechanisms against tumors [20]. However, the cysticercus synthesizes proteins that reduce the production of cytokines IL-2, IL-4, and IFN-*γ* and the recruitment of macrophages by TNF-*α*, thereby rendering Th1 and Th2 immune responses and proinflammatory cytokines inefficient [11, 21]. 

The analyzed cases showed a predominance of Caucasian patients and the same number of female and male patients, which is in agreement with the data described by Del Brutto et al. [19] in their study of patients presenting with glioma and neurocysticercosis.

The NC group exhibited the highest mean age (66 years), which is in agreement with an earlier study that reports a mean age of 44.69 ± 14.04 years in patients with neurocysticercosis and glioma [19]. The aging process contributes to patients' susceptibility to neoplasias, infections, and parasitosis, including cysticercosis [22]. Studies have reported the presence of calcified cysticerci in the brains of most patients with neoplasia, which indicates that the infection precedes the development of the tumor and suggests a temporal relationship between parasitosis and neoplasia [19]. 

In the CC group, the predominance of nonelderly patients (79.7%) is compatible with the literature that describes patients within the age range of 21–40 years as the most affected by cysticercosis [2, 23, 24]. With regard to patients with neoplasia, the mean age reported in the literature is <60 years [25] which was confirmed in the present study.

In the CC and NC groups, the brain was the predominant location, which is consistent with earlier studies [2, 24, 26]. The second most frequent location was the heart. Muscles were the least affected by parasitosis, a finding that contradicts the literature [24]. The fact that an examination of the heart was given precedence over muscle examination during autopsy (with Chagas disease being endemic in the region) could explain the data found in the present study. 

Most patients had 3 or more cysticerci, while the literature reports the predominance of 1 cysticercus (33%–71%). The number, morphologic type, location, development stage of the cysticerci, local immune reactions, and host distance are factors that determine the different individual clinical manifestations. Therefore, there is no pathognomonic sign of the disease [27, 28].

We did not find a correlation between cysticerci location and the system affected by the neoplasia, which supports previous findings [16, 22, 29]. We note that in the study conducted by Del Brutto et al. [19], a correlation was found between neurocysticercosis and glioma.

The fact that the digestive system was the most affected system in the NC and NN groups may be explained by the fact that this system is constantly being damaged by the ingestion of foods containing carcinogenic agents. This finding is in agreement with literature reports postulating that neoplasias of the digestive system are the main cause of morbidity and mortality worldwide [30, 31].

In conclusion, the demographic profile of patients with cysticercosis and neoplasia is similar to that of patients with cysticercosis alone. The location of the cysticerci does not correspond to the location of the neoplasia. Moreover, the incidence of cysticercosis and neoplasia was greater in older patients suggesting that immunosenescence may contribute to development of neoplasia promoted by cysticercosis.

## Figures and Tables

**Table 1:** Demographic profile of autopsied patients with cysticercosis and/or neoplasia from 1970 to 2006.

Groups	Neoplasia and cysticercosis (NC) *n* (%)	Cysticercosis (CC) *n* (%)	Neoplasia (NN) *n* (%)	Without neoplasia and cysticercosis (WW) *n* (%)
Age				
Elderly	8 (100)	52 (78.8)	158 (58.5)	1234 (74.0)
Nonelderly	0 (0)	14 (21.2)	112 (41.5)	434 (26.0)

Ethnic				
Caucasian	7 (87.5)	38 (57.6)	168 (62.2)	1006 (60.3)
Non-Caucasian	1 (12.5)	28 (42.4)	102 (37.8)	662 (39.7)

Gender				
Male	4 (50.0)	44 (66.7)	167 (61.8)	1101 (66.0)
Female	4 (50.0)	22 (33.3)	103 (38.2)	567 (34.0)
